# Smoking Is Correlated to Internal Hernia After Gastric Bypass Surgery: a Post hoc Analysis of Data from a Randomized Clinical Trial

**DOI:** 10.1007/s11695-024-07097-5

**Published:** 2024-02-20

**Authors:** Marlene F. Bossen, Johanne Gormsen, Sara D. Kristensen, Frederik Helgstrand

**Affiliations:** 1 Department of Gastrointestinal and Hepatic Diseases, Surgical Section, Herlev Hospital, Borgmester Ib Juuls Vej 1, 2730 Herlev, Denmark; 2 Department of Surgery, Zealand University Hospital, 4600 Koege, Denmark

**Keywords:** Laparoscopic Roux-en-Y gastric bypass, Internal herniation, Smoking

## Abstract

**Purpose:**

Internal herniation is a well-known complication of laparoscopic Roux-en-Y gastric bypass (L-RYGB). The aim of this study was to evaluate smoking as an independent risk factor for internal herniation after L-RYGB.

**Materials and Methods:**

This study was performed as an exploratory post hoc analysis of data from a previous published randomized controlled trial (RCT) designed to compare closure and non-closure of mesenteric defects in patients undergoing L-RYGB. The primary outcome of this study was to assess the significance of smoking as a risk factor for internal herniation after L-RYGB. Secondary outcome was early postoperative complications defined as Clavien-Dindo grade ≥ 2.

**Results:**

Four hundred one patients were available for post hoc analysis. The risk of internal herniation was significantly higher among patients who were smoking preoperatively (hazard ratio (HR) 2.4, 95% confidence interval (c.i.) 1.3 to 4.5; *p* = 0.005). This result persisted after adjusting for other patient characteristics (HR 2.2, 1.2 to 4.2; *p* = 0.016). 6.0% of the patients had postoperative complications within the first 30 days. 4.9% of these patients were smoking and 6.3% were not smoking, *p* = 0.657. 11.0% of the patients underwent surgery due to internal herniation by 5 years after the primary procedure.

**Conclusion:**

Smoking is a significant risk factor for internal herniation but did not increase risk for 30 days postoperative complications.

**Graphical Abstract:**

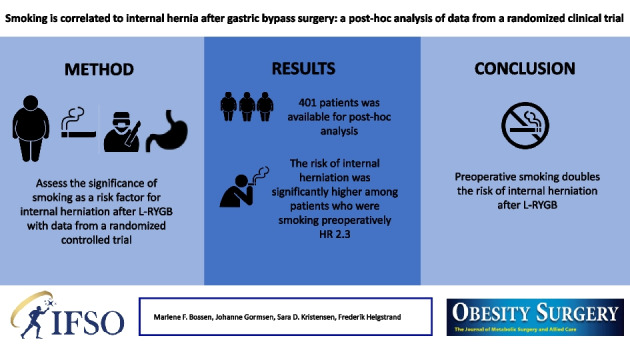

## Introduction

Laparoscopic Roux-en-Y gastric bypass (L-RYGB) is considered the most effective treatment of morbid obesity, resulting in significant weight loss, resolution of obesity-related comorbidities, and improved quality of life [1–3]. When performing L-RYGB, two mesenteric defects are created between the mesentery of the alimentary limb and the mesocolon (Petersen’s space), and between the biliopancreatic limb and the common limb in relation to the entero-enteroanastomosis (mesojejunal defect) [4, 5]. This anatomic alteration may cause small bowel obstruction due to internal herniation of small bowel through these mesenteric defects. The reported incidence of internal herniation ranges from 0.5 to 9% [6]. The most evident risk factor for internal herniation is lack of primary closure of the mesenteric defects [7–9]. Known patient-related risk factors for internal herniation after L-RYGB is greater postoperative weight loss [10] and pregnancy, especially in the third trimester [11].

Smoking increases the risk for postoperative complications after L-RYGB. Smoking is associated with an increased risk of both early complications such as wound complications [12] and late complications such as marginal ulcers [13] among others. According to Danish Health Authorities guidelines, smoking cessation is one of the preconditions prior to hospital referral to evaluation before L-RYGB [14]. Nevertheless, data on the association between smoking and internal herniation is absent. The aim of this study was to evaluate if smoking as an independent risk factor to internal herniation after L-RYGB.

## Methods

### Study Design and Participants

This study was performed as an exploratory post hoc analysis of data from a previous published randomized controlled trial (RCT) designed to compare closure and non-closure of mesenteric defects in patients undergoing L-RYGB [7]. Risk factors for internal herniation found in the study cohort have not previously been published. The primary and secondary outcomes investigated in this study have not been published before.

All patients who underwent L-RYGB surgery during the inclusion period from August 1 2012 to May 18, 2017, at the Zealand University Hospital in Denmark were considered eligible for inclusion. Indication for bariatric surgery followed the criteria from the Danish Health Authorities [15]. Eligibility was assessed in the regional outpatient clinic by a multidisciplinary team consisting of an endocrinologist, a surgeon, a dietician, and a nurse. Candidates for inclusion were informed about the study and were offered enrollment, after which oral and written consent were obtained. Randomization was carried out intraoperatively if, assessed by the primary surgeon after intraabdominal inspection, it was considered possible to perform the procedure laparoscopically. Participants were randomly assigned to either L-RYGB surgery without closure of the mesenteric defects or with primary closure of the mesenteric defects with clips. The schedule for randomization was generated in a 1:1 ratio by block randomization in blocks of 4 participants. The randomization was single blinded until 24 months after the operation. In this retrospective analyses, patients who stated to be smoking at the time of their admission for L-RYGB were defined as being smoking.

### Data Collection

Baseline characteristics including smoking status and intraoperative information were collected prospectively. Postoperative data were collected at scheduled follow-up appointments after 3, 6, 12, and 24 months. Complete follow-up was ensured by cross-checking through nationwide electronic medical records up to 5 years postoperatively

### Outcomes

The primary outcome for this post hoc analysis was to assess the significance of smoking as a risk factor for internal herniation after L-RYGB. Internal herniation was defined as intraoperative identified herniation of the small bowel through one or both of the mesenteric defects requiring surgical reposition of the herniated bowel and closure of the mesenteric defects [7]. Secondary outcome was postoperative complications within 30 days after primary surgery defined as Clavien-Dindo grade ≥ 2 [16].

### Statistical Analyses

Categorical variables were analyzed using the chi-square test. Continuous variables were tested for distribution by plotting of histograms and qq-plots. Normally distributed continuous variables were analyzed using the two-sample *t*-test, otherwise, the Mann-Whitney *U* test.

The Kaplan-Meier estimate was used for the assessment of the time-dependent risk of internal herniation. Survival statistic included the time from the L-RYGB procedure until the end of follow-up on May 19, 2019, or until the first operation for internal herniation. Patients were censored if emigrated or deceased.

A Cox regression model adjusted for sex, age, preoperative body mass index (BMI), preoperative smoking, and weight loss was used to identify independent risk factors for internal herniation.

Results are presented as hazard ratios (HRs) with 95% confidence intervals (c.i.). A *p*-value < 0.05 was considered significant.

Statistical analyses were performed using RStudio version 1.1463 (RStudio, Boston, Massachusetts, USA)

## Results

Four hundred one patients were available for post hoc analysis. Defect closure at the L-RYGB was performed in 201 patients and in 200 patients the mesenteric defect was without closure [7].

All included patients were available for follow-up after 30 days (*n* = 401). After 12 and 24 months, respectively 400 patients (99.8%) and 398 patients (99.3%) were available for follow-up. Median follow-up was 59 months (interquartile range 54–63).

In total, 81 (20.2%) patients were known to be smoking when the L-RYGB was performed. There were no significant differences in baseline characteristics when comparing patients who were smoking versus not smoking preoperatively (see Table 1). However, fewer patients who were smoking 44.4% (*n* = 36) than not smoking 51.6% (165) had mesenteric defect closure, though this was not significant.

**Table 1:** Baseline characteristics

	Overall, *n* = 401	Smoking, *n* = 81	Not smoking, *n* = 320	*p*
Sex				0.068
Female	317 (79.1)	70 (86.4)	247 (77.1)	
Male	84 (20.9)	11 (13.6)	73 (22.8)	
Age (years, median i.q.r.)	41 (34–48)	40 (33–45)	42 (34–49)	0.085
Preoperative BMI (kg/m^2^, median i.q.r.)	44.3 (40.8–49.1)	43.4 (39.2–48.0)	44.6 (41.0–49.2)	0.103
Type 2 diabetes	94 (23.4)	19 (23.5)	75 (23.4)	0.997
Hypertension	117 (29.2)	21 (25.9)	96 (30.0)	0.471
Obstructive sleep apnea	45 (11.2)	10 (10.9)	35 (12.3)	0.720
Polycystic ovarian syndrome	48 (12.0)	7 (8.6)	41 (12.8)	0.302
Arthrosis	104 (25.9)	26 (32.1)	78 (24.4)	0.157
Mesenteric defect closure	201 (50.1)	36 (44.4)	165 (51.6)	0.252

Values are *n* (%) unless otherwise indicated. I.q.r. interquartile range

By 5 years after the primary procedure, 11.0% (*n* = 44) had undergone surgery due to internal herniation. Of these, 36.4% (*n* = 16) of patients who were smoking preoperatively and 63.6% (*n* = 28) of patients not smoking had internal herniation, respectively (see Table 2).

**Table 2:** Risk factors for internal herniation

	Overall, *n* = 401	Internal herniation, *n* = 44	No internal herniation, *n* = 357	*p*	Multivariate HR	Confidence interval	*p*
Sex, *n* (%)				0.758	0.55	0.25–1.23	0.147
Female	317 (79.1)	34 (77.3)	283 (79.3)				
Male	84 (20.9)	10 (22.7)	74 (20.7)				
Age (years, median i.q.r.)	41 (34–48)	40 (32–47)	42 (34–48)	0.193	0.99	0.96–1.03	0.640
Preoperative BMI (kg/m^2^, median i.q.r.)	44.3 (40.8–49.1)	43.4 (41.8–47.3)	44.4 (40.8.49.4)	0.324	1.00	0.94–1.07	0.928
Percentage excess weight loss after 12 months, median (i.q.r)	77.0 (66.9–92.8)	88.1 (71.7–98.6)	76.5 (66.4–92.1)	0.019	1.02	1.00–1.04	0.025
Percentage excess weight loss after 24 months, median (i.q.r)	80.8 (67.9–98.2)	92.6 (71.2–101.8)	80.3 (67.6–97.2)	0.058	-	-	-
Smoking, *n* (%)	81 (20.2)	16 (36.4)	65 (18.2)	0.005	2.20	1.16–4.17	0.016
Closure of mesenteric defects, *n* (%)	201 (50.1)	13 (29.5)	188 (52.7)	0.004	0.39	0.20–0.76	0.005

*I.q.r.*, interquartile range; *HR*, hazard ratio

The risk of internal herniation was significantly higher among patients, who were smoking preoperatively (hazard ratio (HR) 2.4, 95% c.i. 1.3 to 4.5; *p* = 0.005). This result persisted after adjusting for other patient characteristics (HR 2.2, 1.2 to 4.2; *p* = 0.016). Excess weight loss after 12 months was also a significant risk factor to internal herniation (*p* = 0.019). This result also persisted after adjusting for other patient characteristics (HR 1.0, 95% c.i. 1.0 to 1.0; *p* = 0.025). Potential risk factors for internal herniation are presented in Table 2. Neither comorbidities nor postoperative complications within 30 days after primary surgery were significant risk factors for internal herniation. Furthermore, there was a significant association between smoking and weight loss. Patients, who were smoking, had a greater weight loss after both 1 year (*p* = 0.001) and after 2 years (*p* = 0.001).

In total, 6.0% (*n* = 24) patients had a postoperative complication within the first 30 days. Among these patients, 4.9% were smoking and 6.3% were not smoking, *p* = 0.657.

Fourteen patients required reoperation, 2.5% of these patients were smoking, and 3.8% were not smoking. Only one patient, who was not smoking, required treatment at an intensive care unit. No patients died. The total numbers of complications were too low to compare the severity of complications.

## Discussion

This post hoc analysis of data from a RCT showed that smoking more than doubled the risk for later internal herniation but did not increase the risk for 30 days postoperative complications.

A previous retrospective cohort study by Nuytens et al. [17] also identified smoking as a significant risk factor for small bowel obstruction after L-RYGB. However, the association was not significant, when the subgroup was analyzed with differentiation between small bowel obstruction due to internal herniation or adhesions.

Thus, to our knowledge, this is the first study to significantly prove the association between smoking and the risk of intern herniation after L-RYGB.

Smoking was not yet considered an absolute contraindication at the time of this study. However, besides being a risk factor to internal herniation, smoking has previously been associated with multiple both short- and long-term complications to bariatric surgery. This was summarized in a large review by Chow et al. [18], which found smoking to be an independent risk factor for increased 30-day mortality and major postoperative complications, especially wound infections and pulmonary complications. In long term, smoking has been associated with both specific conditions such as marginal ulcers and with generally inferior outcomes such as chronic abdominal pain and lower quality of life [18, 19]. These associations between smoking and development of long-term complications are consistent with the results of the present study. Thus, it might be possible to decrease the incidence of internal herniation along with other complications if eliminating smoking is a risk factor. This requires an extensive effort both pre- and postoperatively from both the patient and medical professionals. Nevertheless, preoperative smoking cessation is now mandatory before L-RYGB according to Danish guidelines [14]. Despite this and the risk of being tested positive in urine cotinine tests, some patients with a history of smoking still smoke when scheduled for L-RYGB [20].

A previous study by Wolvers et al. [21] found that a considerable number of patients continued smoking after bariatric surgery despite recommendations and cessation preoperatively. The patients who continued smoking tended to be less aware of the beneficial effects of smoking cessation after bariatric surgery. A large prospective multi-center study from the USA by King et al. [22] confirmed these results and associated smoking relapse with younger age, low income, marriage, and illicit drug use. Although smoking cessation is recommended before L-RYGB, the prevalence of smoking among patients undergoing bariatric surgery has been reported to be as high as 40% [18]. Therefore, the association found in this study between smoking and the risk of internal herniation after L-RYGB is considered interesting, as smoking is a potentially modifiable risk factor.

The association between excess weight loss and internal herniation is consistent with previous findings by van Berckel et al. [23], and furthermore, smoking was associated with greater weight loss. However, the association between smoking and internal herniation persisted after adjusting for weight loss.

The study has limitations. Foremostly, the study was not designed for the investigated outcomes. On the other hand, a retrospective cohort study is considered to be the most ethical research approach to investigate the consequences of smoking after L-RYGB since it would be unethical, with current knowledge, to continue operate patients who smoke.

The results of the study are based on data on preoperative smoking. Data regarding postoperative smoking in relation to surgery for internal herniation was not available, which introduces a risk of misclassification. However, patients smoking preoperatively are hypothesized to continue smoking postoperatively.

Additionally, the study is missing information on possible confounders including socioeconomic factors and use of illicit drugs, which could influence smoking status. Finally, the study by Wolvers et al. [24] has shown a tendency for underreporting smoking status among candidates for bariatric surgery. This was shown preoperatively but have not yet been investigated postoperatively. This limitation would most likely underestimate the resulting effect of smoking on internal herniation. However, since the study has been performed in a randomized, controlled design with an almost complete long-term follow-up, the results are considered valid.

In the future, patients could benefit from preoperative long-term smoking cessation before being subjected to bariatric surgery. A possible solution required smoking cessation at the time of referral from their general practitioners. Current best practice guidelines recommend 6 weeks of smoking cessation prior to bariatric surgery [25]. However, further knowledge upon the period of smoking cessation and the possible benefits of a longer cessation period is warranted. Furthermore, patients at risk of smoking relapse should be identified preoperatively in order to be able to offer these patients closer follow-up with specific focus on smoking cessation.

In conclusion, preoperative smoking doubles the risk of internal herniation after L-RYGB and this study emphasizes the need for research focusing on intervention strategies for smoking cessation, preventing relapse, and nicotine substitution.
